# Service use and obstetric outcomes in pregnant women attending a Women’s Mental Health Clinic

**DOI:** 10.4102/jcmsa.v4i1.326

**Published:** 2026-04-09

**Authors:** Menasia Patterson, Deidré Mason, Juané Voges, Kerry-Ann Louw

**Affiliations:** 1 Department of Psychiatry, Faculty of Medicine and Health Sciences, Stellenbosch University, Cape Town, South Africa; 2 Department of Obstetrics, Faculty of Medicine and Health Sciences, Stellenbosch University, Cape Town, South Africa

**Keywords:** perinatal mental health, perinatal service use, referral pathways, obstetric outcomes, women’s mental health, perinatal psychiatry

## Abstract

**Background:**

Understanding the characteristics of women attending perinatal mental health services, their service utilisation and obstetric outcomes is important to guide planning and improve perinatal mental health services. This study aimed to describe referral patterns, sociodemographic and clinical profiles, mental health service use and obstetric outcomes among pregnant women attending a tertiary Women’s Mental Health Clinic at an academic tertiary hospital in Cape Town, South Africa.

**Methods:**

A descriptive study of all women who attended the Women’s Mental Health Clinic service from April 2022 to March 2023 (*n* = 82). Pregnant women who attended their first appointment during the sampling time frame (*n* = 67) were then selected for more detailed review.

**Results:**

Most referrals received were for managing pre-existing mental illness in pregnancy (72.0%, *n* = 59), with most women attending their first Women’s Mental Health appointment in the second trimester (58.2%, *n* = 39). Referrals were largely from the hospital’s high-risk obstetric clinic (61.0%, *n* = 50). Mood disorders, including depression (43.3%, *n* = 29) and bipolar disorder (17.9%, *n* = 12), were the most common diagnoses. Psychotropic medications were used in 80.6% of the sample (*n* = 54). The rates of substance use (35.8%, *n* = 24) were lower compared to other South African studies of pregnant women attending specialised maternal mental health services. Referral to and uptake of allied mental health services were variable.

**Conclusion:**

The findings underscore the need for earlier identification and referral of women with perinatal mental health conditions, improved service integration and monitoring of obstetric outcomes.

**Contribution:**

This study provides context-specific knowledge that may inform clinical care, funding and future research directions for pregnant women attending mental health clinics.

## Introduction

Pregnancy represents a unique period in a woman’s life, characterised by a myriad of physical and psychological changes. Women known with mental health conditions can experience exacerbation or relapse of symptoms in pregnancy, and many women experience poor mental health for the first time during pregnancy.^1^ External circumstances or other health conditions can place some women at greater risk for poor mental health during pregnancy.^1^ This may include adolescent pregnancy, poverty, previous difficult birth experiences, physical health conditions, having inadequate support, unwanted pregnancy or substance abuse.^1^

Perinatal mood and anxiety disorders represent the most prevalent mental health conditions among women of reproductive age.^2^ Prevalence estimates of common perinatal mental disorders (CPMDs) vary greatly across contexts, in part because of differences in study methodology, including how researchers define the perinatal period, and whether they use screening or diagnostic data to estimate prevalence.^3^ In high-income countries, the burden of perinatal depression is estimated to be between 10% and 20%.^4^ In contrast, in low- and middle-income countries (LMICs), the prevalence of perinatal depression is 24.7%.^4^

Perinatal mental health conditions have wide-ranging consequences for women, their children and the community at large, including intergenerational poverty, women’s morbidity and mortality and children not meeting growth and development indicators.^5^ A meta-analysis looking at antenatal depression and its association with adverse birth outcomes in LMICs, published in 2020, found that a history of antenatal depression was associated with a 59% higher risk of adverse obstetric outcomes.^6^ These outcomes included preterm labour and low birth weight (LBW).^6^

There is an urgent need to implement evidence-based, community-driven perinatal mental health interventions, with changes to national policy and prioritisation in budgetary allocations. The World Health Organization (WHO) advocates for a stepped-care approach to perinatal mental health.^1,2,7^ According to this approach, women should receive care according to the degree of their mental health concerns. This tiered care model delivers low-intensity, evidence-based interventions to the majority of individuals, reserving the most resource-intensive interventions for those with the most severe or complex mental health needs.^1,2,3^ Perinatal mental health services should include health promotion, prevention, early detection and intervention, treatment and follow-up of perinatal mental disorders.^5^ A recent case study demonstrated the successful implementation of a stepped-care maternal mental health service model in South Africa.^7^ To improve women’s mental health services, intersectoral collaboration with government departments, the health sector, social development departments, non-governmental organisations (NGOs), community-based services and patients and their families is essential for ensuring adequate mental health of all pregnant women.^5^

Understanding the characteristics of women attending perinatal mental health services, their service utilisation and obstetric outcomes is important to guide planning of perinatal mental health services and implementation of evidence-based strategies to improve services. This is particularly important in resource-constrained settings. This study aimed to describe referral patterns and to evaluate the sociodemographic characteristics, clinical profile, mental health service use and obstetric outcomes of pregnant women attending a tertiary Women’s Mental Health Clinic in Cape Town, South Africa.

## Research methodology and design

### Study design

This cross-sectional, descriptive study included a retrospective folder review of women who attended a Women’s Mental Health Clinic between 01 April 2022 and 31 March 2023.

### Study setting

The study was conducted at Tygerberg Hospital (TBH), a tertiary, academic hospital, in Cape Town, South Africa. The socioeconomic status of the referral area is mainly low- to middle-income communities, with high rates of unemployment and poverty. Referrals to TBH are mostly from primary care and secondary hospitals located within the referral area.

The Women’s Mental Health Clinic at TBH was established in 2017 to provide specialised women’s mental health care, including perinatal mental health care. The clinic runs once per week and is run by a transdisciplinary team including a consultation-liaison psychiatrist, trainee psychiatrist, senior clinical psychologist and nursing staff, with social worker and occupational therapist input on a referral basis. A weekly mentalisation group for pregnant and post-partum women and their babies is facilitated by a clinical psychologist on the same day as the clinic. The clinic accepts referrals of women’s mental health-related disorders not responding to primary care interventions. Indications for referral include mental illness during the perinatal period (up to 12 months post-partum), pregnancy planning for women known with psychiatric illness and mental health symptoms related to infertility, pregnancy loss, menopause, gynaecological cancers, termination of pregnancy, chronic pelvic pain and premenstrual dysphoric disorder.

### Study population and sampling strategy

A convenience sampling method was used. All women referred to the Women’s Mental Health Clinic who attended the clinic between 01 April 2022 and 31 March 2023 were included in the study. Pregnant women who attended their first appointment during the sampling period were then selected for a more detailed description of their clinical and demographic characteristics, service utilisation and obstetrics outcomes. Women were followed up at the clinic for up to 1-year post-partum. A sampling end date of 31 March 2023 allowed for the description of the outcomes of all pregnant women referred during the study period.

### Data collection

Data were extracted from hospital folders and the TBH electronic record-keeping systems and entered into the Research Electronic Data Capture (REDCap) system. The reasons for referral and referral source were recorded for all women attending their first appointment during the study period. Patient folders of all pregnant women who attended their first appointment during the study period were selected for further detailed folder review. Data extracted included:

**Demographic characteristics at the time of the first consultation:** Age, marital status, highest level of education, employment status and number of living biological children.

**Clinical characteristics at the time of the first consultation:** Reason for and source of referral, gravidity, parity, current pregnancy gestation, previous pregnancy complications, psychiatric diagnoses, current substance use and medical comorbidities. Psychiatric diagnoses were made by a specialist psychiatry consultant or specialist psychiatry trainee using the Diagnostic and Statistical Manual of Mental Disorders (DSM-5-TR) criteria.^8^ For patients in whom the psychiatric diagnoses changed over the assessment period, the diagnoses at the last clinical contact were documented.

**Service utilisation up until 1-year post-partum, discharge from the clinic or loss to follow-up:** Number of psychiatry appointments attended, referrals to social work, psychology and occupational therapy services and number of appointments attended, psychiatric admissions and psychotropic medications used (existing use at time of first consultation and psychotropic medication initiated at any time during service utilisation). Valproate and other antiepileptics were counted as psychotropic medications if prescribed for bipolar disorder and not if prescribed for epilepsy.

**Obstetrics outcomes:** Antenatal complications, method of delivery, delivery complications and neonatal complications, including LBW and prematurity. Low birth weight was defined as a birth weight of less than 2500 grams, and prematurity was defined as less than 37 completed weeks of pregnancy.^9^

### Data analysis

Data was analysed using Stata Version 18.1 ([computer program]. College Station, TX: StataCorp LLC, 2023). Continuous variables were summarised as mean and standard deviation, while nominal variables were summarised as counts and percentages.

### Ethical considerations

Ethical clearance to conduct this study was obtained from the Faculty of Medicine and Health Sciences, Stellenbosch University Research Ethics Committee (No.: S23/10/241) and approval from the National Health Department and management of TBH (NHRD reference: WC_202311_044). A waiver of informed consent was granted. All data were anonymised to ensure the privacy and confidentiality of participants’ personal information.

## Results

Of the 82 women who attended the clinic, 81.7% (*n* = 67) were referred for assessment and management of mental illness during the pregnancy state, while 18.3% (*n* = 15) were referred for assessment and management of mental illness outside of the pregnancy state (Table 1). Monitoring of existing mental illness was the most frequent reason for referral during pregnancy (72.0%, *n* = 59), while post-partum mental illness (8.5%, *n* = 7), followed by chronic pelvic pain (3.7%, *n* = 3), were the most common reasons for referral in non-pregnant women. Only 2 women (2.4%) were referred for pregnancy planning management. Most referrals were from the TBH High-Risk Obstetric Clinic (61.0%, *n* = 50).

**TABLE 1:** Reason and source of referral for all participants attending the Women’s Mental Health Clinic (*N* = 82).

Variables	*n*	%
**Referral source**	-	-
TBH high-risk obstetric clinic	50	61.0
TBH consultation-liaison services	8	9.8
Community clinic	9	11.0
TBH psychiatry outpatient services	9	11.0
TBH psychiatry inpatient services	2	2.4
District hospitals	2	2.4
Private health services	2	2.4
**Referrals during pregnancy**	67	81.7
Existing mental illness in pregnancy for monitoring	59	72.0
New onset of mental illness in pregnancy	6	7.3
Existing mental illness for relapse review	1	1.2
Evaluation of possible mental illness in pregnancy	1	1.2
**Referrals for non-pregnant women**	15	18.3
Post-partum mental illness	7	8.5
Chronic pelvic pain	3	3.7
Planning pregnancy with mental illness	2	2.4
Mental illness related to menopause	2	2.4
Mental illness related to gynaecological cancer	1	1.2
Premenstrual dysphoric disorder	0	0.0
Mental illness related to miscarriage or termination of pregnancy	0	0.0
Mental illness related to infertility	0	0.0

TBH, Tygerberg Hospital.

All participants self-identified as women in this sample. Most women were in the 30–39 years age category (46.3%, *n* = 31), and married (34.3%, *n* = 23) or in a relationship (46.3%, *n* = 31) (Table 2). There was only one adolescent pregnancy (1.5%) managed during the study period. A large number of women had secondary level education (77.6%, *n* = 52), but most were unemployed (70.1%, *n* = 47). While gestation at first visit spanned all trimesters, most women attended their first appointment in the second trimester (58.2%, *n* = 39). Gravidity ranged from first pregnancy (23.9%, *n* = 16) to multiple pregnancies, with almost a quarter (23.9%, *n* = 16) of women having been pregnant five or more times. Grand multiparity (five or more deliveries) was only seen in two women (3.0%), with 23.9% (*n* = 16) of the women having three or more deliveries. In this sample, 40.2% (*n* = 27) of women had experienced previous pregnancy complications, with miscarriage being the most common pregnancy complication (28.4%, *n* = 19).

**TABLE 2:** Demographic and clinical characteristics of pregnant women (*N* = 67).

Variables	*n*	%
**Age category (years)**		
Younger than 18	1	1.5
18–29	26	38.8
30–39	31	46.3
40–49	9	13.4
**Relationship status**		
Single	11	16.4
Married	23	34.3
In a relationship	31	46.3
Separated	2	3.0
Divorced or widowed	0	0.0
**Highest level of education**		
Primary school education	4	6.0
Secondary school education	52	77.6
Tertiary education	11	16.4
**Employment**		
Employed	20	29.9
Unemployed	47	70.1
**Gestation at the time of first visit (weeks)**		
0–12	6	9.0
13–27	39	58.2
> 27	22	32.8
**Gravidity**		
1	16	23.9
2	15	22.4
3	13	19.4
4	7	10.4
5 and more	16	23.9
**Parity**		
0	21	31.3
1	16	23.9
2	14	20.9
3	7	10.4
4	7	10.4
5 or more	2	3.0
**Previous pregnancy complications**		
Yes	27	40.2
No	29	43.3
Not disclosed	11	16.4
**Type of pregnancy complications and/or outcomes**		
Miscarriage	19	28.4
Preterm labour < 37 weeks	5	7.5
Termination of pregnancy†	4	6.0
Placental abruption	1	1.5
FGR	0	0.0
LGA	0	0.0
Other	5	7.5

FGR, foetal growth restriction; LGA, large for gestational age.

† , Termination of pregnancy was included as a previous pregnancy complication and/or outcome, as it represents a clinically important pregnancy outcome in this population; the reason for termination of pregnancy was not documented.

Major depressive disorder (43.3%, *n* = 29) was the most common psychiatric diagnosis in this sample, followed by the category ‘Other’ (19.4%, *n* = 13). Twelve (17.9%) women had bipolar disorder and six (9.0%) had schizophrenia (Table 3). The category ‘Other’ included intellectual disability (9.0%, *n* = 6), psychosis secondary to another medical condition (7.5%, *n* = 5), obsessive compulsive disorder (1.5%, *n* = 1) and specific phobia (1.5%, *n* = 1). In this sample, 80.6% (*n* = 54) of pregnant women used psychotropic medications. Antidepressants were the most common class of psychotropic used (43.3%, *n* = 29), followed by oral antipsychotics (37.3%, *n* = 25) and mood stabilisers (4.5%, *n* = 3) (including lithium and antiepileptics). No pregnant woman used sodium valproate or benzodiazepines in this study sample. Only one participant (1.5%) was on a long-acting injectable antipsychotic. In this cohort, 35.8% (*n* = 24) of women reported using substances in pregnancy, while only one patient was diagnosed with a substance use disorder (1.5%) and two patients were diagnosed with a substance-induced mood or psychotic disorder (3%). Nicotine was recorded as the most used substance (28.4%, *n* = 19), with alcohol use being recorded in only 4.5% (*n* = 3) of women.

**TABLE 3:** Psychiatric diagnoses, substance use and psychotropic medication use in pregnant women (*N* = 67).

Variables	*n*	%
**Psychiatric illness†**		
Major depressive disorder	29	43.3
Bipolar disorder	12	17.9
Generalised anxiety disorder	9	13.4
Schizophrenia	6	9.0
Personality disorders	5	7.5
Adjustment disorder	3	4.5
Panic disorder	3	4.5
Trauma-related disorders	3	4.5
Substance-induced psychotic disorder or substance-induced mood disorder (SIPD or SIMD)	2	3.0
Feeding and eating disorder	2	3.0
Substance use disorder	1	1.5
Somatic symptom and related disorders	1	1.5
Neurocognitive disorder	0	0.0
Attention-deficit hyperactivity disorder (ADHD)	0	0.0
Other	13	19.4
**Psychotropic medication uses** ‡		
Yes	54	80.6
No	15	22.4
**Class of psychotropic medication**		
Antidepressants	29	43.3
Antipsychotics	25	37.3
Mood stabilisers (includes lithium and anti-epileptic agents)	3	4.5
Long-acting antipsychotic injectable	1	1.5
Other	1	1.5
Benzodiazepines	0	0.0
**Substance use**		
No	43	64.2
Yes	24	35.8
**Type of substance use**		
Nicotine	19	28.4
Alcohol	3	4.5
Caffeine	1	1.5
Methamphetamine	1	1.5
Cocaine or cannabis or heroin	0	0.0

† , Some women had more than one specified diagnosis;

‡ , Some women were on more than one psychotropic medication, which included existing use at the time of consultation and psychotropic medication initiated at any time during service utilisation.

In this group of participants, 44.8% (*n* = 30) of women had medical comorbidities, the most common being hypertension (22.4%, *n* = 15) (Table 4). The most common non-psychotropic medication reportedly used was haematinics (37.3%, *n* = 25).

**TABLE 4:** Medical diagnoses and management in pregnant women (*N* = 67).

Variable	*n*	%
**Medical condition**		
Hypertension	15	22.4
Diabetes	5	7.5
Epilepsy	2	3.0
Gestational diabetes	1	1.5
Autoimmune disorders	1	1.5
Other	15	22.4
**Non-psychotropic medication**		
Haematinics†	25	37.3
Anti-hypertensives	14	20.9
Anti-diabetic treatment	6	9.0
Anti-epileptic treatment	2	3.0
Other	12	17.9
**Referral to another medical discipline**		
No	60	89.6
Yes	7	10.4
**Medical disciplines referred to**		
High-risk obstetrics	3	4.5
Neurology	2	3.0
Internal medicine	0	0
Surgery	0	0
Genetics	0	0
Other	2	3.0

† , The National Integrated Maternal and Perinatal Care Guidelines for South Africa recommend that all pregnant women be prescribed haematinics to prevent anaemia. As haematinics are dispensed directly at clinics, this may not be recorded in the folder or pharmacy records.^10^

In this cohort, 41.8% (*n* = 28) of pregnant women attended only one psychiatrist appointment, while the majority attended two or more (Table 5). Of the women who only attended one appointment, 10.7% were discharged, while 89.3% were lost to follow-up. Five women (7.5%) required hospital admission for inpatient psychiatric care. All admissions occurred during pregnancy, with women admitted at gestations spanning all three trimesters. This included three voluntary and two involuntary admissions under the Mental Health Care Act (No. 17 of 2002). In this sample, 70.1% (*n* = 47) of pregnant women were referred for group or individual psychotherapy, while of those referred, 70.2% (*n* = 33) attended at least one appointment. A total of 25.4% (*n* = 17) of pregnant women attended the mentalisation group; most of those women attended only one to two group sessions (70.6%, *n* = 12). Of the women who attended individual psychotherapy, 58.3% (*n* = 14) attended only one to two sessions. Among women included in this study, 19.4% (*n* = 13) of pregnant women were referred to social services and 13.4% (*n* = 9) for occupational therapy interventions.

**TABLE 5:** Mental healthcare service utilisation of pregnant women (*N* = 67).

Variable	*n*	%
**Number of Women’s Mental Health Clinic psychiatry appointments attended**		
1	28	41.8
2	13	19.4
3	9	13.4
4	4	6.0
5 and more	13	19.4
**Psychiatric admission required (voluntary or involuntary)**		
No	62	92.5
Yes	5	7.5
**Psychology (individual and group therapy)**		
Referred	47	70.2
Referred and attended†	33	70.2
Referred and did not attend†	14	29.8
Not referred	20	29.9
**Number psychology sessions (individual therapy)** ‡		
1–2	14	58.3
3–4	6	25.0
5–6	2	8.3
7–8	1	4.2
More than 8	1	4.2
**Number of psychology sessions (group therapy)§**		
1–2	12	70.6
3–4	4	23.5
5–6	1	5.0
**Social worker**		
Referred	13	19.4
Referred and attended¶	12	92.3
Referred and did not attend¶	1	7.7
Not referred	54	80.6
**Occupational therapy**		
Referred	9	13.4
Referred and attended††	3	33.3
Referred and did not attend††	6	66.7
Not referred	58	86.6

† , Percentage of total number referred (*n* = 47);

‡ , Percentage of total number attending individual therapy (*n* = 24);

§ , Percentage of total number attending group therapy (*n* = 17);

¶ , Percentage of total number referred (*n* = 13);

†† , Percentage of total number referred (*n* = 9).

The most frequent antenatal complication reported was abnormal cardiotocography (CTG) of various causes (55.2%, *n* = 37), followed by pre-eclampsia (23.9%, *n* = 16) and preterm labour (10.4%, *n* = 7) (Table 6). Only one woman in this sample had a termination of pregnancy (for social reasons). In terms of method of delivery, 46.3% (*n* = 31) had an uncomplicated normal vaginal delivery (NVD), 10.4% (*n* = 7) had an uncomplicated planned elective caesarean section (CS) and 28.4% (*n* = 19) had an emergency CS. Other delivery complications included perineal tears (10.4%, *n* = 7) and instrumental vaginal delivery (3.0%, *n* = 2). Low birth weight and prematurity were encountered in 10.4% (*n* = 7) and 9.0% (*n* = 6) of live births, respectively.

**TABLE 6:** Obstetrics and neonatal outcomes in women attending a Women’s Mental Health Clinic (*N* = 67).

Variables	*n*	%
**Previous antenatal complications and outcomes**		
Abnormal CTG	37	55.2
Pre-eclampsia	16	23.9
Preterm labour	7	10.4
Termination of pregnancy†	1	1.5
FGR	1	1.5
Placental abruption	0	0.0
LGA	0	0.0
Miscarriage	0	0.0
**Uncomplicated delivery outcomes** ‡		
Normal vaginal delivery	31	46.3
Planned caesarean section	7	10.4
**Complicated delivery outcomes**		
Emergency caesarean section	19	28.4
Perineal tears	7	10.4
Assisted delivery (vacuum and forceps deliveries)	2	3.0
Shoulder dystocia	1	1.5
Uterine rupture	1	1.5
Post-partum haemorrhage	0	0.0
Other	4	6.0
**Neonatal complications§**		
Low birth weight	7	10.4
Prematurity	6	9.0
LGA	2	3.0
Stillbirth	0	0.0
Congenital malformations	0	0.0
Sepsis	0	0.0
Other	2	3.0

CTG, cardiotocography; FGR, foetal growth restriction; LGA, large for gestational age.

† , Termination of pregnancy was included as a previous pregnancy complication and outcome, as it represents a clinically important pregnancy outcome in this population; the reason for the termination of pregnancy was not documented;

‡ , Eight cases of missing data affecting delivery outcomes;

§ , Eight cases of missing data affecting neonatal complications.

## Discussion

This study aimed to describe a detailed profile, including referral patterns, service use and outcomes for pregnant women attending a tertiary Women’s Mental Health service. Several important findings were identified. Firstly, most of the referrals were for the assessment and management of pre-existing mental illness in pregnancy and were sourced internally from the hospital’s high-risk obstetric clinic. Most pregnancy-related referrals attended the first appointment in the second trimester of pregnancy. Secondly, mood disorders were the most common psychiatric diagnoses in pregnancy, the majority of women used psychotropic medication, and the self-reported substance use was lower than found in other South African studies. Thirdly, patterns of referral to and engagement with allied health services were variable. Finally, the incidence of LBW and preterm birth in this sample was lower when compared to local and other African studies.

This Women’s Mental Health Clinic functions predominantly as a gestational mental health service, with most women attending their first mental health appointment in the second trimester of pregnancy. Very few women were referred for pregnancy planning, and reproductive planning is often overlooked in the mental health care of women with both common mental disorders (CMDs) (e.g. depression, anxiety) and serious mental illnesses (SMIs) (e.g. schizophrenia, bipolar disorder). Current National Institute for Health and Care Excellence (NICE) guidelines encourage discussion with all women of childbearing age, with co-occurring mental illness, around reproductive planning and contraceptive use.^11^ South African guidelines highlight mental health issues as part of preconception care, but do not make specific recommendations regarding referral to specialised maternal health services preconception.^10^ In LMICs, barriers to pregnancy planning consultations and family planning services include fragmented health systems, limited mental health services and sociocultural norms that restrict women’s autonomy in pregnancy and family planning.^11^ While grand multiparity was only seen in two women, almost a quarter of the women reported having three or more deliveries, and contraceptive counselling may not be optimal in this population. Women with high parity may have greater caregiving burdens that contribute to mental distress, while nulliparous women with a psychiatric history may require more intensive perinatal support.^11^ It is important to examine how mental health influences reproductive decision-making and outcomes, including unintended pregnancies or suboptimal birth spacing. Early detection, with regular screening of women during their pregnancy at any contact with health and social services, ensures early referral and treatment for women at risk.^12,13^ Integrated obstetric-psychiatric care models and proactive family planning discussions are thus recommended.

Most referrals were from the hospital’s high-risk obstetric clinic. Although it is positive that there is a close relationship between the high-risk obstetrics and the Women’s Mental Health Clinic, it is concerning that referrals from community clinics were few. The predominance of internal referrals suggests missed opportunities for external providers, such as general practitioners, midwives and community health workers, to identify and refer patients not responding to primary care interventions. This could be attributed to inconsistent implementation of screening protocols, uncertainty about referral pathways at the time of the study and stigma associated with mental health.^11^ While the clinic has a detailed referral pathway document, with high staff turnover at primary care facilities, this information may not always be accessible. Updated South African guidelines recommend resource mapping as part of comprehensive maternal care; this includes a detailed listing of support and referral resources.^10^ Increasing awareness and regular identification of referral pathways at the community clinic level are recommended.

Most women were first seen at the clinic during their second trimester. In the context of LMICs, late referrals to perinatal mental health services are frequently linked to systemic barriers such as lack of trained healthcare personnel, under-resourced primary healthcare settings and absence of routine screening during early antenatal care visits.^14^ Updated South African guidelines recommend mental health screening at least once at the time of pregnancy booking and, if there are adequate resources, further screening once per trimester and during the postnatal period (6 weeks to 3 months).^10^ The previous 2016 guidelines, applicable at the time of this study, did not include a chapter on maternal mental health or specific mental health screening guidelines, although psychiatric history was recommended as part of antenatal history taking.^10^ Unplanned pregnancy and seeking antenatal care late during pregnancy may also be factors to consider. Early identification in the first trimester remains uncommon because of limited antenatal engagement, especially among socioeconomically disadvantaged populations. The first trimester is a critical window for identifying and intervening in mental health conditions that could impact both maternal and foetal health.^15^ The integration of task-sharing strategies by training community health workers in perinatal mental health screening and the introduction of community-based mental health awareness campaigns to reduce stigma can all assist with earlier mental health service engagement during pregnancy.^16^ Early screening and detection of mental health concerns not responding to primary care interventions, with early referral to specialised health services, is recommended to assist with a positive pregnancy experience and decrease adverse obstetric and neonatal outcomes.

Worldwide, more than 16 million adolescent women (15–19 years) give birth each year, with more than 50% in sub-Saharan Africa.^17^ In South Africa, the weighted pregnancy prevalence was 22% in the 15–19 years age category in a 2020 systematic review and meta-analysis.^18^ Adolescent mothers may fall into the gap between child and adolescent and adult health services, including perinatal mental health services.^19^ Adolescents represent a highly vulnerable group with distinct psychosocial challenges, including higher risks of unplanned pregnancy, socioeconomic disadvantage and lack of support systems.^11,19,20^ The low referral rates in this sample may reflect barriers such as reduced access to services, service management done at the primary or district level, lack of adolescent-specific care models and concerns about confidentiality.^11^ We recommend the establishment of adolescent-specific care models to assist in managing this vulnerable group.

Mood disorders were the most common psychiatric diagnoses made in pregnant women, including major depressive disorder and bipolar disorder. The predominance of depression and anxiety in the clinic’s sample is consistent with broader epidemiological trends, where CMDs are more prevalent among perinatal populations.^11^ However, the underrepresentation of SMIs like schizophrenia raises important questions. One explanation may be that these individuals are more likely to be managed in tertiary psychiatric settings and less likely to engage with perinatal-specific services.^15^

Although substance use was reported by more than a third of women, very few had a formal substance use disorder diagnosis. The rates of reported substance use in the sample are lower than those in other South African studies of pregnant women.^21^ A recent retrospective review of 399 pregnant women attending an antenatal clinic in Johannesburg, South Africa, found a prevalence rate of substance use of 45%.^22^ Similarly, a recent retrospective study of pregnant women attending specialised maternal health services in Cape Town, South Africa, found higher rates of alcohol (16.8%), methamphetamine (5.9%), cannabis (4.0%) and tobacco (34.7%) use.^21^ Overall, substance use disorders appear to be underdiagnosed in pregnancy.^23^ This may stem from underreporting by patients who fear judgement, stigma or involvement of social services, and some women with substance use disorders do not seek antenatal care at all.^23^ There may be hesitance among clinicians to broach sensitive topics without sufficient support.^23^ Furthermore, clinicians may lack adequate training or time to conduct comprehensive assessments, particularly in busy antenatal clinics. This highlights the need for incorporating standardised tools for screening SMI and substance use disorders.^24^ Recommendations include strengthening partnerships with perinatal mental health and addiction services for this vulnerable population and training staff on sensitive and effective substance use inquiry and harm reduction strategies.

In this cohort, the majority of women used psychotropic medications during pregnancy, with antidepressants being the most reported. These findings are comparable with a recent South African study, which found that 95% of women attending two specialised maternal mental health services in Cape Town, South Africa, were prescribed psychotropics at any point during their pregnancy. The most prescribed psychotropic in these participants was non-tricyclic antidepressant (64%).^21^ The high use of antidepressants may also be indicative of the fact that the clinic receives referrals of patients who have not responded to primary care interventions. Encouragingly, no women were prescribed sodium valproate, aligning with international safety recommendations. Antipsychotic use in our sample may reflect a clinical population with significant symptom burden. However, this also raises questions about whether pharmacological interventions are being prioritised above non-pharmacological therapies.^11,25^ Recommendations include allocation of increased resources in the form of allied health staff and services to this specialised Women’s Mental Health Clinic. Of note is that the reported use of haematinics was low, given that national guidelines recommend use in all pregnant women to prevent anaemia.^10^ As haematinics are dispensed directly at the clinics, this may not be reported in the hospital records.

While psychology services had a relatively high referral rate, referral rates to social work and occupational therapy were considerably lower, which is concerning. Multidisciplinary care is a cornerstone of perinatal mental health, and poor service utilisation may indicate systemic access issues, long waitlists or poor integration between services.^19^ Patients may also lack knowledge of the benefits of these interventions or may be disinclined to attend multiple appointments. In some cases, allied health services may be inaccessible because of financial or logistical barriers. Lack of knowledge among healthcare practitioners about the referral pathways and allied health services available could be another reason why there was low allied health service engagement, particularly occupational therapy and social worker services. Healthcare providers may adopt a medication-centric approach, which can limit attention to alternative treatment strategies. This highlights an important disparity in access or utilisation across different allied health services and suggests that engagement may be dependent on referral practices and patient uptake.

The findings in this study suggest that treatment adherence may be a more immediate concern. Although the referral rate to psychology services was high, the majority of women attended only one or two sessions. Similarly, most of those who attended psychiatry appointments did so only once or twice, suggesting that sustained engagement with services is limited. This pattern may reflect difficulties related to continuity of care, competing social demands or system-level barriers such as appointment scheduling and follow-up. Advances in technology have played a significant role in addressing gaps within healthcare systems, with alternative models such as task-sharing,^16^ peer support^16^ and virtual care^26,27^ showing promise in overcoming some of these challenges. In particular, digital health interventions – including telemedicine and mental health applications – have expanded service accessibility and strengthened treatment possibilities.^26^ Yet, this also raises concerns about safety and confidentiality, and further research is required.^26^ This study did not directly assess these interventions or explore reasons for disengagement. Future research should focus on understanding the factors contributing to low adherence and explore whether these newer service delivery models can improve both initial uptake and sustained engagement with care. Furthermore, clear referral pathways, improved education about services and enhanced provider training remain essential for improving the overall mental healthcare continuum.

The rates of LBW and preterm births are lower in this sample than those reported in broader South African population-based studies. This is perhaps because of the small sample. A cohort study conducted in KwaZulu-Natal reported LBW and preterm delivery rates of 13.5% and 16.4%, respectively.^9^ A large-scale United Kingdom study analysing over 2 million pregnancies found that women with prior engagement with mental health services were significantly more likely to deliver preterm (1 in 10) compared to those without such contact (1 in 15).^27^ Similarly, evidence from LMICs indicates that antenatal depression is associated with a 1.2-fold increased risk of preterm birth and a 1.3-fold increased risk of LBW,^6^ highlighting the global impact of maternal mental health on perinatal outcomes. A Zimbabwean study that examined the association between antenatal depression and adverse birth outcomes found an association between antenatal depression and LBW, but no other adverse obstetric outcome was found to be statistically significant.^28^ An Ethiopian study conducted by Beyene and colleagues found that women with antenatal depression were 2.51 times more likely to have a child with LBW.^29^ However, direct comparisons must be made cautiously, considering differences in sample characteristics and study design. Close monitoring, early identification of those at risk and early interventions to mitigate the risk of adverse obstetric outcomes in pregnant women with co-occurring mental illness are recommended.

The CS rate in this sample was comparable to the South African public hospital CS rate of 32.3%.^30^ By comparison, a 2020 study from KwaZulu-Natal examining delivery methods in women with peripartum depression reported that 53.3% of participants had NVDs, and 46.7% underwent CS.^31^ Among those who had CS, 51.1% were elective and 48.8% were emergency procedures.^31^ Global CS rates have risen substantially, with 21.1% of women delivering by CS between 2010 and 2018, and projections indicate further increases by 2030.^32^ In LMICs, this trend exposes a complex triad of challenges: an unmet need for medically indicated CS, elevated procedural risks and growing concerns around the unnecessary overuse of CS, particularly within contexts marked by significant health disparities.^32^ Safe, evidence-based and consensual birth practices for all women with mental illness are recommended for a positive birthing experience.

Women in this study reported high rates of previously experienced miscarriage. A Norwegian study found that women with a history of mental illness were at increased risk of miscarriage, while women who experienced miscarriage were more likely to develop mental health disorders.^33^ These findings further emphasise the importance of integrated mental health and obstetric care, particularly in settings where both psychiatric morbidity and adverse pregnancy outcomes are both prevalent and under-recognised.

The study had several limitations. The small sample size and study setting limit the generalisability of the findings to broader populations of pregnant women. As a descriptive study, no causal relationships could be inferred between observed variables. The small sample size also limited the investigation of associations between variables. The study did not record the use of traditional, complementary and alternative medications, which are important to explore because pregnant women access a large spectrum of interventions. Finally, findings were limited by missing data and reporting bias, particularly for self-reported mental health and substance use history. Additional research with larger, more diverse samples and analytical designs powered to compare outcomes in different groups is needed to explore and expand upon these findings.

## Conclusion

This study offers valuable insights into the characteristics and service use patterns of pregnant women attending a specialised Women’s Mental Health Clinic at a tertiary hospital. By focusing on a specific and often underrepresented population, the study adds context-specific knowledge that may inform clinical care, funding and future research directions. Strengthening early detection and routine screening for depression, anxiety and substance use during antenatal visits should be supported to facilitate timely referral and treatment. Embedding mental health screening tools within maternal health assessments and electronic health records can promote systematic identification of women at risk, including adolescent pregnancy. Improved integration between obstetric and mental health services is essential. Integrating mental health professionals within obstetric clinics, developing clear referral pathways and training primary care and community health workers can enhance coordination, continuity and accessibility of care. Expanding multidisciplinary collaboration through greater access to allied health services – such as psychology, occupational therapy and social services – would support holistic and family-centred care. Training clinicians in culturally sensitive and trauma-informed approaches may further improve outcomes. The use of digital and telehealth interventions offers promising opportunities to address service gaps, particularly in under-resourced areas, although careful attention to privacy and ethical standards is required. Finally, investment in perinatal mental health infrastructure and workforce development, alongside the creation of national guidelines tailored to local contexts, is critical to ensure equitable, standardised and sustainable care.
